# Complete ileal transection: A rare complication of adhesive bowel disease—A case report

**DOI:** 10.1016/j.ijscr.2019.03.002

**Published:** 2019-03-20

**Authors:** Qurrat Al Ain Atif

**Affiliations:** H. No. 473, St. 62, G-9/4, Islamabad, Pakistan

**Keywords:** Adhesive bowel disease, Acute small bowel obstruction, Ileal transection, Postoperative adhesions

## Abstract

**Introduction:**

Adhesive bowel disease is a sequel of abdominal surgeries and has come forth as the leading cause of small bowel obstruction (SBO). It may also lead to chronic abdomino-pelvic pain and infertility in women. Adhesions form as a result of peritoneal irritation, giving rise to an inflammatory process. The treatment of choice for adhesive small bowel obstruction remains controversial, and relies on the surgeon’s judgment.

**Case presentation:**

Being described is an unusual case of a young girl who developed complete ileal transection secondary to postoperative adhesive band formation.

**Conclusions:**

Adhesive small bowel disease (ASBD) and small bowel obstruction are a major cause of patient distress and financial and clinical practice burden. It is usually a diagnosis of exclusion and leads to delayed treatment and morbidity. Many preventive techniques and materials are being tested to prevent adhesion formation. A lot more needs to be done to prevent, in the first place, the formation of adhesions or treat this condition to reduce such morbid outcomes as strangulation or transection of gut.

## Highlights

• Post-operative adhesions are common after abdomino-pelvic surgery.
• Adhesions have been named the commonest cause of intestinal obstruction.
• Various risk factors for formation of adhesions.
• Halstedian principles to be followed to minimize risk of post-operative adhesions.

## 1 Introduction

Acute small bowel obstruction, previously seen only with incarcerated hernia, is now attributed majorly to postoperative adhesions as an increasing number of abdominal surgeries are being performed for varying etiologies [1]. As much as 60–70% of intestinal obstructions are caused by adhesions [2,3]. Adhesions form in 63–97% of patients undergoing major abdominal surgery [3,4] Adhesive small bowel obstruction (ASBO) is a major cause of readmissions, accounting for 4.5% of readmissions in the first year following surgery [4]. According to data published around 20–40% are treated surgically whereas remainder being managed conservatively [1].

ASBO is a leading cause of surgical admissions (16%) 5 and due to tendency to recur, it markedly increases morbidity and health care costs [1]. ASBO can lead to mortality in as many as 3% in simple obstruction to 30% in cases of strangulation [1].

Bands formed as a result can be so tight as to case ischemia and necrosis of the affected small bowel segment leading to complete transaction as with our patient [6]. Such a case has been reported once before, to the best of our knowledge.

## 2 Case presentation

**Fig. 1:**
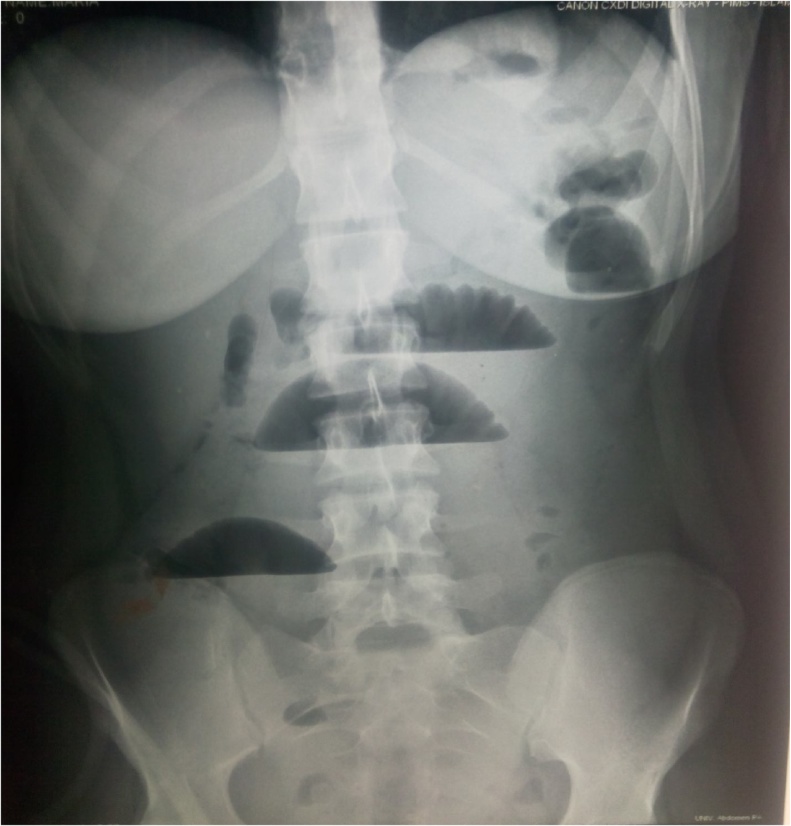
Erect x ray abdomen showing dilated bowel loops.

The following case is being reported in line with the SCARE criteria [7]. An 18-year old Pakistani girl presented to the emergency department of a tertiary care hospital, with recurrent abdominal pain and persistent non-bilious vomiting for the past 04 days. Upon presentation she was hemodynamically stable and her abdominal examination was unremarkable. Her blood workup was normal. Only her erect radiographs showed dilated small bowel loops with air-fluid levels (Fig. 1). Ultrasonography of abdomen revealed thick walled distended small bowel loops with to and fro movement, suggesting intestinal obstruction.

She gave a history of an abdominal surgery at the age of 2 years, of whose nature the parents and the patient were unaware and no documentation was available. Patient was not on any medication and family history was unremarkable. Moreover, patient has had multiple recurrent admissions to the hospital for intestinal obstruction in the past 6 months, which all resolved with conservative management. Patient was of the view that her symptoms are the result of the surgery she had during her childhood.

**Fig. 2:**
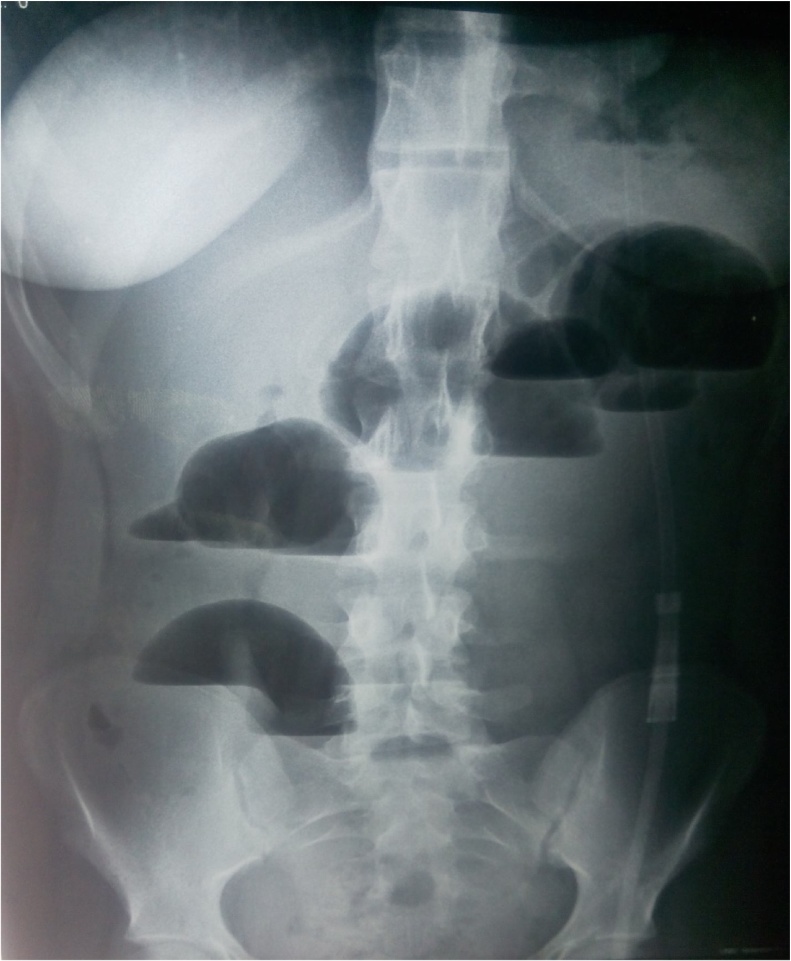
Abdominal x ray at day 2.

As before, she was put on conservative management with nasogastric decompression and intravenous fluid therapy. Her condition failed to resolve as a radiograph two days later appeared to have even worse dilatation of bowel loops (Fig. 2).

**Fig. 3:**
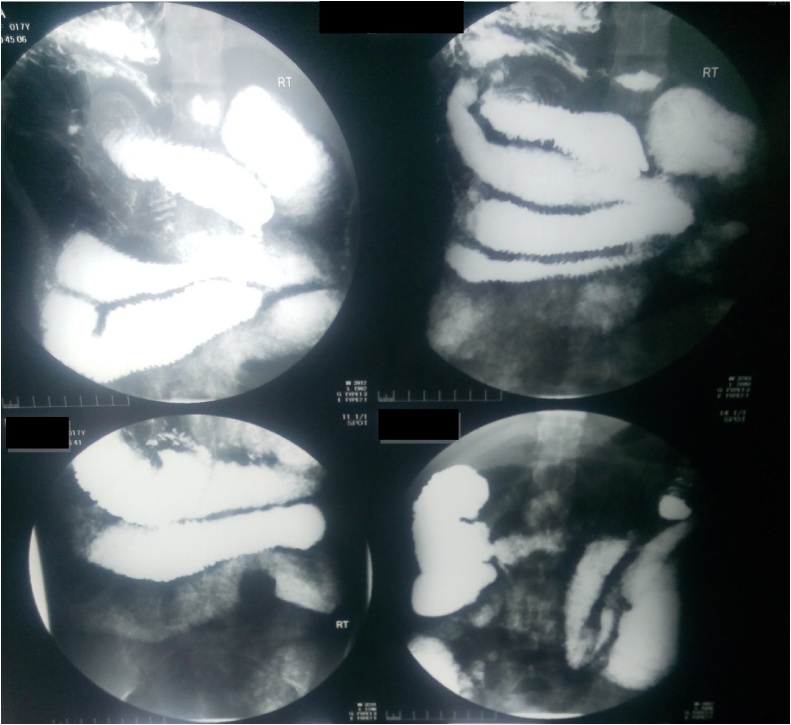
Barium meal and follow through.

**Fig. 4:**
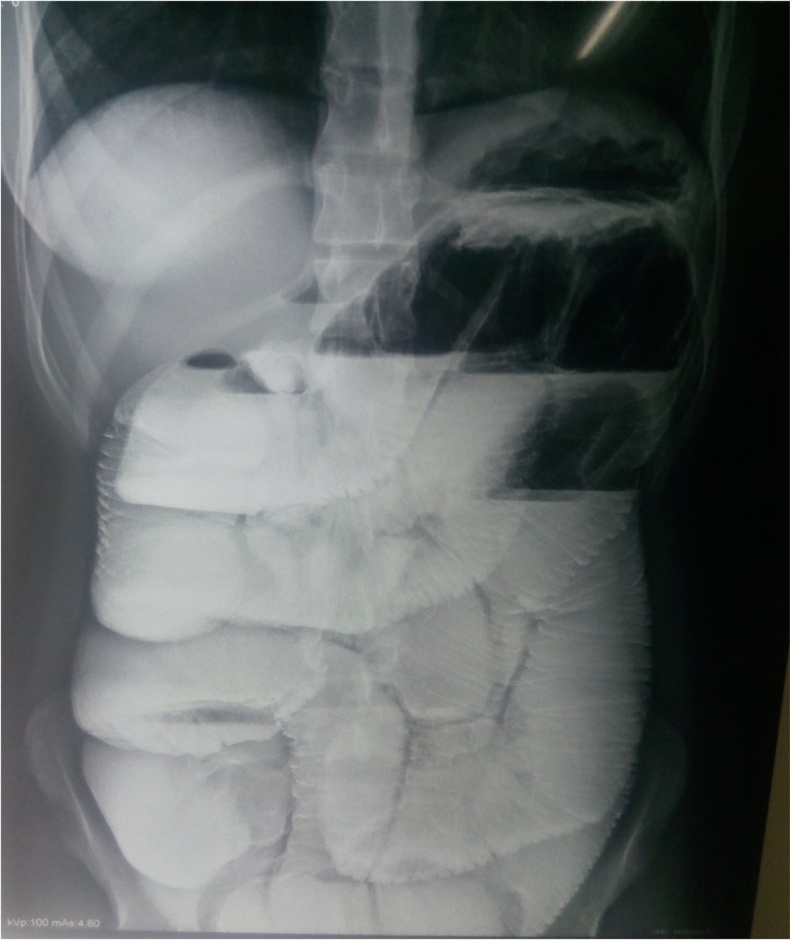
Post barium meal x ray abdomen.

Barium meal follow through revealed thickened folds and dilatation of small bowel (Fig. 3, Fig. 4). Decision was made to explore her for possible adhesions and adhesiolysis.

**Fig. 5:**
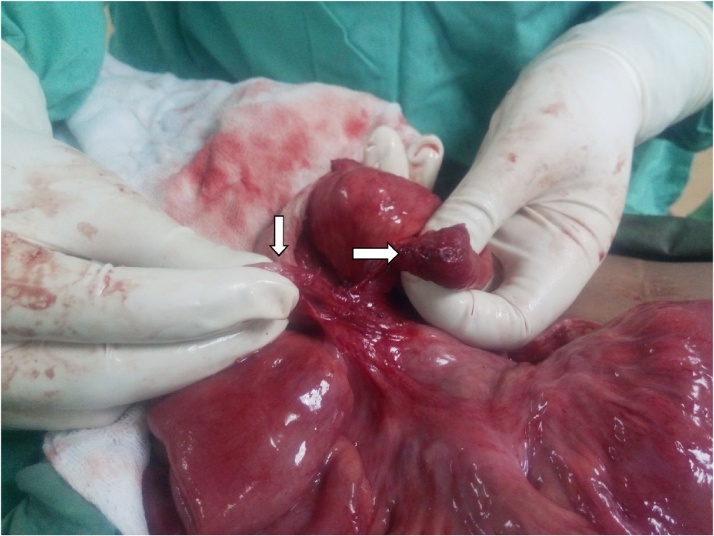
Blind ending loops of ileum (White arrows).

**Fig. 6:**
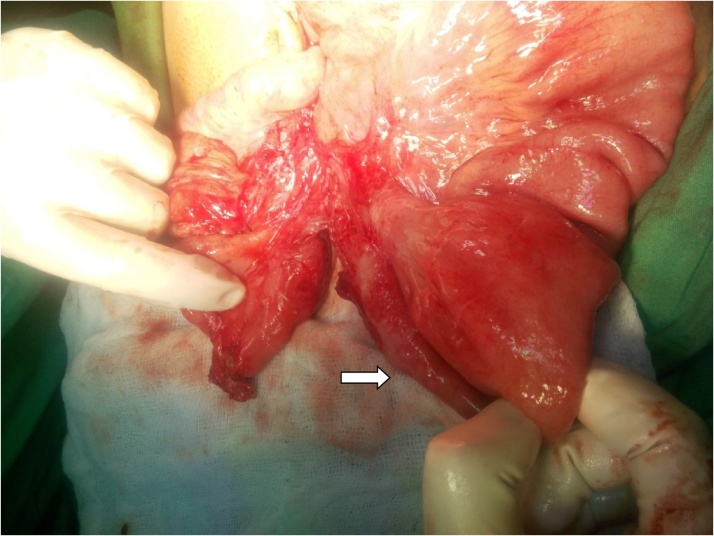
completely transected ileum (white arrow).

Surgery was performed by assistant professor of surgery at the hospital. Per-operatively, a band was identified at 2 feet distal to duodeno-jejunal junction. Upon further exploration, two blind ending loops of ileum were found buried in the band (Fig. 5, Fig. 6). Stapled anastomosis was done with uneventful post-operative course in surgical ward.

Histopathology was consistent with an ileal band and the patient is symptom free at 4 months post-operatively.

## 3 Discussion

Small bowel obstruction is a condition where there is interruption of forward flow of intestinal contents. It was first described by Hippocrates and first treatment was performed by Praxagoras in 350 BC [6]. Bowel obstruction makes up 16% of all surgical admissions [5].

Leading cause of small bowel obstruction is adhesions (60%), followed by hernia (25%) and neoplasm (5–10%) [2,3]. First adhesions were identified at post-mortem of a patient in 1836 [4].

Adhesions are pathological bonds between omentum, bowel loops and abdominal wall [4]. These might contain thin connective tissue, thick fibrous bridge with blood vessels and nerves or might be direct contact between two organ surfaces [4]. Adhesions may be congenital or acquired (post inflammatory or post-operative). Post-operative adhesions form at surgical site, non-surgical site or after adhesiolysis, these three processes identified as adhesion formation, de novo adhesion formation or adhesion reformation, respectively [4]. Another classification divides adhesions into 2 classes; type I (de novo) or type II (adhesion reformation) both further sub classified as A or B [4].

Adhesions form in 93–100% of cases after upper abdominal surgeries and 67–93% of lower abdominal surgeries [8]. Adhesions form most commonly after appendectomies, colorectal and gynecological procedures, reason being free movement of small bowel in the pelvis [9]. Other complications of post operative adhesions include chronic abdominal or pelvic pain and infertility [4,10]. 65–90% of patients with adhesive bowel obstruction have had one or more surgeries [1]. One study demonstrated previous surgery rates as 32.4% colorectal, 27.8% upper abdominal, 19.9% gynecological, 8.5% middle abdominal, 5.1% abdominal wall, 4.5% urological and 1.7% unknown procedures in patients presenting with ASBO [1]. After abdominopelvic surgery, one third patients were readmitted for either adhesion-related complications or re-intervention, out of which >20% were during first post-operative year and 4.5% for ASBO [4]. Although, ASBO has been reported as far as ten years post-operative in about 20% patients [2].

Fortunately, only 15–18% of these patients require second surgery [8]. Laparoscopic approach significantly reduces the risk of adhesion formation by 45% [8].

Adhesions may even form a tight constricting band around the bowel, leading in time to complete transection and formation of two blind loops [6], as was the case with our patient.

Mortality rates for ASBO range from 3% for simple obstruction to a massive 30% with bowel necrosis or perforation [2].

Risk factors for ASBO including type of initial surgery, site of adhesion, timing and recurrence rate of adhesive obstruction haven’t been fully understood [2]. Factors favoring formation of adhesions include anastomotic site, raw areas after serosal tears, trauma, talc, gauze and silk suture [6]. Although laparoscopic approach is considered to promote less adhesions but duration of CO_2_ pneumoperitoneum and insufflation pressures have been linked to increased adhesion formation [4].

Adhesion prevention involves general principles and surgical techniques, mechanical barriers and chemical agents [4].

Adhesions can be prevented by following basic Halstedian principles, which should be applied to all surgical procedures [4]. Careful tissue handling, meticulous hemostasis, continuous irrigation and avoiding unnecessary drying, minimizing use of foreign bodies like suture, gauze, talc, and use of atraumatic clamps reduce risk for adhesion formation [4].

Addition of 2–4% oxygen during laparoscopic procedure reduces adhesion formation, indicating peritoneal hypoxia as underlying cause [4].

Several liquid and solid barriers have been tested with varying results. Liquid barriers include crystalloids, dextran, hyaluronic acid, cross-linked hyaluronic acid and icodextran. Solid barriers such as oxidized regenerated cellulose, expanded polytetrafluoroethylene, hyaluronic acid-carboxymethylcellulose and polyethyleneglycol have been shown to be of benefit.

Chemical agents prevent fibrin organization. Commonly NSAIDs, corticosteroids, calcium channel blockers, histamine antagonists, antibiotics, fibrinolytic agents, anticoagulants, antioxidants, hormones, vitamins, colchicines and selective immune-suppressors have been used and found effective. Local anesthetic agents, owing to their anti-inflammatory properties have also been shown to inhibit adhesion formation [4].

A detailed history, including history of prior surgeries and thorough physical examination are paramount to the diagnosis of bowel obstruction. ASBO is commonly a diagnosis of exclusion [3]. Among many diagnostic modalities, computerized tomography (CT) readily identifies the site, level and cause of obstruction [3]. It has a sensitivity of 81–94% and a specificity of 96% in diagnosing ASBO [1].

Gastrograffin is used to categorize patients for non-surgical management of bowel obstruction. It successfully identified 91% of patients for conservative management in a study [11].

Treatment of ASBO is either conservative or surgical. Studies have reported that 60–80% of the patients are treated conservatively while 20–40% undergo surgery but the choice and timing of management option is still debatable. Bologna guidelines prefer a non-operative approach in absence of strangulation, since re-intervention promotes further adhesion formation. Observation period varies in different studies from 72 h to as long as 10 days [1]. Although, conservatively treated patients have higher recurrence rates and shorter time to readmission [1]. Usefulness of gastrograffin in identifying the need for intervention was determined by a study which demonstrated failure of gastrograffin to reach colon within 24 h as an indication for surgery [10]. Apart from that, gastrograffin has a therapeutic role also. It decreases bowel wall edema and increases bowel motility [11].

Moreover, persistent abdominal pain, fever, tachycardia, abdominal tenderness, rebound tenderness and muscular resistance indicate strangulation, in which situation surgical intervention should be undertaken [1].

Formation of post-operative adhesions is a common complication. It is the leading cause of small bowel obstruction and can lead to such dreadful conditions as complete bowel transaction. The physician and patient should be aware of possibility of adhesions and it should clearly be mentioned in the informed consent. Etiology, pathophysiology, diagnostic modality and choice of treatment option are not fully understood. Prevention is better than cure holds true in the setting of adhesions.

## Conflicts of interest

None.

## Sources of funding

None.

## Ethical approval

Yes approval form has been attached.

## Consent

Informed written consent was taken and identity not disclosed.

## Author’s contribution

Data collection, literature search and manuscript writing was done by Dr. Qurrat Al Ain Atif, who also assisted the surgery.

## Registration of research studies

N/A.

## Guarantor

Dr. QUrrat Al Ain Atif is the guarantor of submission.

## Provenance and peer review

Not commissioned, externally peer reviewed.

## References

[1] Eren T., Boluk S., Bayraktar B., Ozemir I.A., Yildirim Boluk S., Tombalak E. (2015). Surgical indicators for the operative treatment of acute mechanical intestinal obstruction due to adhesions. Ann. Surg. Treat. Res..

[2] Ellis H. (1997). The clinical significance of adhesions: focus on intestinal obstruction. Eur. J. Surg. Suppl..

[3] Sinwar P.D. (2015). Small bowel obstruction secondary to greater omental encircling band-Unusual case report. Int. J. Surg. Case Rep..

[4] Arung W., Meurisse M., Detry O. (2011). Pathophysiology and prevention of postoperative peritoneal adhesions. World J. Gastroenterol..

[5] Marc Neff M.D., Brian Schmidt D.O. (2010). Laparoscopic treatment of a postoperative small bowel obstruction. JSLS.

[6] Liaqat N., Dar S.H. (2013). Transection of gut loop due to post-operative adhesions. APSP J. Case Rep..

[7] Agha R.A., Fowler A.J., Saetta A., Barai I., Rajmohan S., Orgill D.P., the SCARE Group (2016). The SCARE statement: consensus-based surgical case report guidelines. Int. J. Surg..

[8] Ouaissi M., Gaujoux S., Vevrie N., Deneve E., Brigand C., Castel B. (2012). Post-operative adhesions after digestive surgery: their incidence and prevention: review of the literature. J. Vise Surg..

[9] Evers B.M., Townsend M.C., Beauchamp R.D., Evers B.M., Mattox K.L. (2011). Small bowel. Sabiston Textbook of Surgery: The Biological Basis of Modern Surgical Practice.

[10] ten Broek R.P., Issa Y., van Santbrink E.J., Bouvy N.D., Kruitwagen R.F., Jeekel J. (2013). Burden of adhesions in abdominal and pelvic surgery: systematic review and met-analysis. BMJ.

[11] Wadani H.A., Al Awad N.I., Hassan K.A., Zakaria H.M., Abdulmohsen Al Mulhim A., Alaqeel F.O. (2011). Role of water soluble contrast agents in assigning patients to a non-operative course in adhesive small bowel obstruction. Oman Med. J..

